# T Helper 9 Cells: A New Player in Immune-Related Diseases

**DOI:** 10.1089/dna.2019.4729

**Published:** 2019-10-07

**Authors:** Jing Chen, Lian Guan, Lin Tang, Shiming Liu, Ya Zhou, Chao Chen, Zhixu He, Lin Xu

**Affiliations:** ^1^ Special Key Laboratory of Gene Detection and Therapy of Guizhou Province, Zunyi, Guizhou, China.; ^2^ Department of Immunology, Zunyi Medical University, Zunyi, Guizhou, China.; ^3^ Department of Medical Physics, Zunyi Medical University, Zunyi, Guizhou, China.; ^4^ Key Laboratory of Adult Stem Cell Transformation Research, Chinese Academy of Medical Sciences, Zunyi, Guizhou, China.; ^5^ Department of Pediatrics, Affiliated Hospital of Zunyi Medical University, Zunyi, Guizhou, China.

**Keywords:** Th9 cell, tumor, IL-9, inflammation

## Abstract

The helper T cell 9 (Thelper-9, Th9), as a functional subgroup of CD4^+^T cells, was first discovered in 2008. Th9 cells expressed transcription factor PU.1 and cytokine interleukin-9 (IL-9) characteristically. Recent researches have shown that the differentiation of Th9 cells was coregulated by cytokine transforming growth factor β, IL-4, and various transcription factors. Th9 cells, as a new player, played an important role in various immune-related diseases, including tumors, inflammatory diseases, parasite infection, and other diseases. In this article, we summarize the related research progress and discuss the possible prospect.

## Introduction

The plasticity of CD4^+^ T cells is critical to the induction of immune response in a context-dependent manner, which reflects the complexity of the connection among different CD4^+^T cell subgroups. Th9 cell (helper T cell 9) is a new subgroup of CD4^+^T cells, which can be differentiated from initial CD4^+^T cells induced by transforming growth factor β (TGF-β) and interleukin-4 (IL-4), and secretes IL-9 characteristically. Th9 cell was named by Veldhoen (Veldhoen *et al.*, 2008) in 2008. Cytokine TGF-β and IL-4 were documented critical for the differentiation of Th9, which were called “Th9 differentiation conditions.” In mice, Th9 cells mainly secrete IL-9 and IL-10. However, human Th9 cells mainly secrete IL-9, and do not secrete IL-10 (Ma *et al.*, 2010).

In recent years, Th9 cells and their related functional factors have been found to play important roles in inflammatory diseases, autoimmune diseases, tumors, and other related clinical diseases, which displays the important role of CD4^+^T cell plasticity in the development of immune-related diseases.

## The Differentiation of Th9 Cells

### Cytokines and membrane molecules

As mentioned above, many cytokines, including TGF-β and IL-4, are involved in the differentiation of Th9 cells. Veldhoen *et al.* (2008) first observed that Th2 cells could be transformed into Th9 cells induced by TGF-β alone. Further studies have shown that Th2 cells could secrete both IL-4 and IL-9 at the early stage of cell differentiation. However, in the presence of TGF-β, these cells further differentiated into Th9 cells, rather than Th2 cells (Takami *et al.*, 2012). Interestingly, Dardalhon *et al.* (2008) found that the initial CD4^+^T cells could also be induced into Th9 cells under the TGF-β and IL-4 conditions. Consistently, Veldhoen *et al.* isolated naive CD4^+^T cells from TGF-β signaling deficiency (TGF-β receptor II knockout) mice and cultured these cells in the presence of TGF-β and IL-4, and then found that cells could not be induced into Th9 cells.

As for mechanism, one study has revealed that TGF-β-Smad2/4 signaling regulates IL-9 expression by displacement of EZH2 and removal of suppressive H3K27 histone modification at the IL-9 locus (Wang *et al.*, 2013). These data suggested that TGF-β plays a key role in the induction of Th9 cells.

Further studies showed that many cytokines were also involved in the differentiation of Th9 cells. On the one hand, some of these cytokines, such as IL-21 and tumor necrosis factor-α (TNF-α), play positive roles in the differentiation of Th9 cells. Th9 cells can secrete cytokine IL-21. In addition, Ma (Ma *et al.*, 2010) and others found that IL-21 could effectively promote the Th9 cells to secrete IL-9, thus forming positive feedback on its differentiation and function. Recently, Jiang *et al.* (2019) further found that TNF-α could promote Th9 cell differentiation, survival, and proliferation through TNFR2-STAT5 signaling pathway and NF-κB signaling pathway. In addition, Anuradha *et al.* (2016) have found that the increased proportion of Th9 cells is closely related to the levels of IL-10 and TGF-β in serum in chronic worm infection, and this phenomenon can be reversed after antiworm treatment, suggesting that IL-10 is also involved in the generation of Th9 cells.

However, the underlying mechanism still needs to be elucidated in depth. On the other hand, other cytokines, such as Interferon-γ (IFN-γ), have opposite ability in Th9 cell differentiation. For instance, Murugaiyan *et al.* (2012) found that IFN-γ could inhibit Th9 differentiation and the secretion of IL-9, both *in vitro* and *in vivo*, which depended on the production of IL-27 from dendritic cells (DCs). Even though the exact mechanism remains to be elucidated, this research work further indicated that there was the coordinative effect of different cytokines in Th9 differentiation.

Besides the above cytokines, the membrane molecules expressed by CD4^+^T cells are also involved in the differentiation of Th9 cells. OX40 (TNF receptor super family 4, TNFRSF4) is a T cell costimulatory molecule, belonging to the TNF receptor super family. The interaction between OX40 and its ligand OX40L, expressed on the antigen-presenting cell (APC) membrane, can induce the transduction of CD4^+^T cell-activated costimulatory signal and play an important role in T cell-mediated immune response (Croft, 2010; Massa and Seliger, 2017). Recent studies have found that OX40/OX40L interaction plays an important role in the overexpression of IL-9 in CD4^+^T cells (Xiao *et al.*, 2012; Kaplan *et al.*, 2015). In terms of mechanism, Xiao *et al.* (2012) found that the OX40/OX40L axis promoted the differentiation and IL-9 expression of Th9 cells by activating the NF-κB pathway.

In addition, Toll-like receptor 2 (TLR2) as documented could promote the differentiation of Th9 cells in the presence of TGF-β and IL-4 (Karim *et al.*, 2017). Also, TNF-like ligand 1A (TL1A) was also reported to elevate Th9 cell differentiation and function (Tsuda *et al.*, 2019). In conclusion, various cytokines and membrane molecules are involved in the differentiation of Th9 cells (as shown in Table 1), which reflects the complexity of differentiation among different CD4^+^T cell subsets and the plasticity of CD4^+^T cell population.

**Table 1.** Related Molecules Involved in Th9 Cell Differentiation

*Molecules*	*Functions*	*References*
TGF-β, IL-4	Promote the differentiation of Th9 cells	Veldhoen *et al.* (2008), Dardalhon *et al.* (2008)
TNF-α	Promote Th9 cell differentiation, survival, and proliferation	Jiang *et al.* (2019)
IFN-γ	Inhibit Th9 differentiation and the secretion of IL-9	Murugaiyan *et al.* (2012)
OX40/OX40L axis	Promote the differentiation and IL-9 expression of Th9 cells by activating the NF-κB pathway	Xiao *et al.* (2012), Kaplan *et al.* (2015)
TLR2	Promote the differentiation of Th9 cells	Karim *et al.* (2017)
TL1A	Promote Th9 cell differentiation and function	Tsuda *et al.* (2019)
GITR	Promote the production of Th9 cells	Kim *et al.* (2015), Xiao (2015)

GITR, ligating glucocorticoid-induced TNFR-associated protein; IFN-γ, interferon-γ; IL, interleukin; OX40, TNF receptor super family 4; TGF-β, transforming growth factor β; Th9, the helper T cell 9; TL1A, TNF-like ligand 1A; TLR2, Toll-like receptor 2; TNF-α, tumor necrosis factor α.

## Transcription Factors Related to the Differentiation of Th9 Cells

### PU.1

It has been well documented that PU.1 is a specific transcription factor for Th9 cells (Ramming *et al.*, 2012; Li *et al.*, 2014). Studies have shown that PU.1-deficient mice have low expression of IL-9, however, upregulated PU.1 endowed Th9 cells with IL-9 secretion in large quantities (Chang *et al.*, 2010). At the same time, the effect of TGF-β in Th9 cell differentiation depended on the activation of Smad pathway and the expression of PU.1 (Elyaman *et al.*, 2012). In terms of mechanism, some studies showed that PU.1 could directly bind to the core region of IL-9 promoter and promote the differentiation of Th9 cells by chromatin modification (Chang *et al.*, 2010; Goswami *et al.*, 2012). In addition, PU.1 could also interfere with the GATA-binding protein 3 (GATA3) and then inhibit the differentiation of Th2 cells and the expression of other subtypes of Th2 cells (Jabeen *et al.*, 2013).

### Signal transduction and transcription activator 6 and GATA3

Recent studies have shown that the expression level of IL-9 in signal transduction and transcription activator 6 (STAT6) and GATA3-deficient mice reduced significantly, which suggested that STAT6 and GATA3 play importance roles in Th9 cell differentiation (Goswami *et al.*, 2012; Perumal and Kaplan, 2012). It was further shown that the expression level of GATA3 increased significantly during the differentiation of Th9 cells and gradually disappeared when Th9 cell matured (Dardalhon *et al.*, 2008; Veldhoen *et al.*, 2008). Studies on the mechanism have shown that GATA3 did not regulate the differentiation of Th9 cells directly, but indirectly participated in the differentiation of Th9 cells by downregulating the level of Foxp3 (Goswami *et al.*, 2012). In the meantime, this process was regulated by the transcription factor PU.1 (Hadjur *et al.*, 2019).

In other words, GATA3 inhibited the differentiation of CD4^+^T cells into regulatory T (Treg) in the early stage, whereas PU.1 regulated the differentiation of Th9 in the later stage. Finally, STAT6, as an important factor for IL-4 signaling pathway, has similar function in the differentiation of Th9 cells. Under the condition of STAT6 deficiency, the initial CD4^+^T cells could not differentiate into Th9 cells in the presence of IL-4 and TGF-β, but had higher expression level of Foxp3 (Chapoval *et al.*, 2010). These studies suggested that STAT6 and GATA3 may be involved in the differentiation of Th9 cells by inhibiting the expression of Foxp3 and the production of IL-9 (Mengyao *et al.*, 2018). However, the relationship between STAT6 and PU.1 still needs to be clarified.

### IFN regulatory factor 4

IFN regulatory factor 4 (IRF4) can be induced by TCR signal, and it is also involved in the differentiation of Th2 and Th17 cells (Ahyi *et al.*, 2009). Meanwhile, IRF4 is documented as a newly discovered transcription factor of Th9 cells, and CD4^+^T cells with IRF4 gene deficiency cannot differentiate into Th9 cells (Staudt *et al.*, 2010). The mechanisms of IRF4 regulating IL-9 expression in Th9 cells are complex, including: IRF4 coordinated the regulation of IL-9 expression with the basic leucine zipper transcription factor (BATF), but this pathway depended on the regulation of STAT6 (Jabeen *et al.*, 2013; Huber and Lohoff, 2014). At the same time, IRF4 could also regulate the differentiation of Th9 cells by means of EICE binding to PU.1 and synergism with TGF-β-signaling molecule smad2 and smad3 protein (Ebel and Kansas, 2016).

### Forkhead protein 1

Recent studies have shown that forkhead protein 1 (Foxo1) was involved in the differentiation of Th9 cells and the secretion of IL-9. The transcription function of Foxo1 was affected by its phosphorylation (Calnan and Brunet, 2008), which reduced in Th9 cells compared with Th0 and Th2 cells (Malik *et al.*, 2017), suggesting that the transcription function of Foxo1 in Th9 cells is stronger than that in Th0 and Th2 cells. Mechanistic aspect, Foxo1 regulates the differentiation of Th9 cells by interacting with various transcription factors. For example, Foxo1 could directly bind to the IL-9 promoter of induced Treg (iTreg), Th17, Th2, and Th9 cells, and promote these cells to express IL-9 (Malik *et al.*, 2017; Buttrick *et al.*, 2018). Moreover, Foxo1 deletion could reduce the expression of PU.1 in Th9 cells, indicating that Foxo1 might affect the differentiation of Th9 cells by regulating PU.1. In addition, Foxo1 could also bind to IRF4 and activate IRF4, thus promoting the differentiation of Th9 cells (Staudt *et al.*, 2010).

### B lymphocyte-induced maturation protein 1

The transcriptional repressor B lymphocyte-induced maturation protein 1 (Blimp-1) has a key role in terminal differentiation in T cell subtypes. For instance, previous studies have found that Blimp-1 can inhibit Th1 cell differentiation (Cimmino *et al.*, 2008) and participant in Tfh cell (Johnston *et al.*, 2009) and CD8^+^T cell differentiation (Kallies *et al.*, 2009). Interestingly, most recent evidence has shown that Blimp-1 can repress the Th9 differentiation program and IL-9 production. Moreover, the inhibition of Blimp-1 by other cytokines related to Th9 cell differentiation is essential for high IL-9 production of Th9 cells (Benevides *et al.*, 2019), which indicates that there are some negative regulators of Th9 programming, even though the exact mechanism remains to be elucidated.

**FIG. 1.**
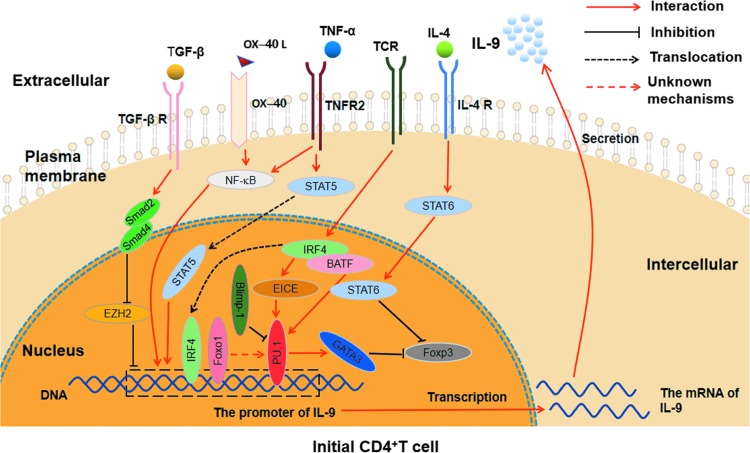
The related cytokine signaling pathways and transcription factors involved in Th9 cell differentiation. PU.1 can directly combine with the IL-9 promoter region and enhance the inhibition of GATA3 on Foxp3; IRF4, which is induced by TCR signaling, regulates the differentiation of Th9 cells through combining with IL-9 promoter region and promoting the interaction between EICE and PU.1, as well as coregulating with BATF and STAT6; Foxo1 affects the differentiation of Th9 cells by combining with the IL-9 promoter region and indirectly regulating PU.1; Blimp-1 represses the Th9 cell differentiation and IL-9 production through binding to PU.1. TGF-β-Smad2/4 signaling regulates IL-9 expression by displacement of EZH2 to promote the differentiation of Th9 cells; IL-4 signaling promotes Th9 differentiation by activating STAT6 to repress Foxp3; TNF-α influences Th9 cell differentiation through TNFR2-STAT5 signaling pathway and NF-κB signaling pathway; OX40/OX40L axis affects the differentiation and IL-9 expression of Th9 cells by activating the NF-κB pathway. BATF, basic leucine zipper transcription factor; Blimp-1, B lymphocyte-induced maturation protein 1; GATA3, GATA-binding protein 3; IL, interleukin; IRF4, interferon regulatory factor 4; NF-κB; STAT, signal transduction and transcription activator; TGF-β, transforming growth factor β; Th9, the helper T cell 9.

Taken together, there are a variety of transcription factors, cooperating with each other, involved in the regulation of Th9 cell differentiation (as shown in Table 2 and Fig. 1).

**Table 2.** Th9 Cells and Their Related Transcription Factors

*Transcription factor*	*Functions*	*References*
PU.1	Specific transcription factor for the differentiation of Th9 cells	Chang *et al.* (2010), Ramming *et al.* (2012), Jabeen *et al.* (2013)
GATA3, STAT6	Regulate the differentiation of Th9 cells by downregulating the level of Foxp3	Chapoval *et al.* (2010), Goswami *et al.* (2012), Mengyao *et al.* (2018)
IRF4	Regulate the differentiation of Th9 cells	Staudt *et al.* (2010), Jabeen *et al.* (2013), Huber and Lohoff (2014), Ebel and Kansas (2016)
FOXO1	Regulate the differentiation of Th9 cells by interacting with various transcription factors	Staudt *et al.* (2010), Malik *et al.* (2017), Buttrick *et al.* (2018)
Blimp-1	Repress Th9 cell differentiation program and IL-9 production	Benevides *et al.* (2019)

Blimp-1, B lymphocyte-induced maturation protein 1; FOXO1, forkhead protein 1; GATA3, GATA-binding protein 3; IRF4, IFN regulatory factor 4; STAT6, signal transduction and transcription activator 6.

## Th9 Cells and Clinical Diseases

### Th9 cells and tumors

Recent researches have shown that Th9 cells play a dual role in tumorigenesis, including their effects on immune cells and tumor cells. Chemokine receptor 6 (CCR6) is mainly expressed by Langerhans cells, memory T cells, and B cells (Ouyang *et al.*, 2012). Th9 cells can stimulate epithelial cells by IL-9 to produce CCR6 ligand CCL20, which recruits immature DCs (iDCs) and other APCs into tumor region (Shumin *et al.*, 2015), and then these APCs present antigen to activate CD8 cytotoxic T lymphocyte (CTLs) to kill tumor cells (Rumbo *et al.*, 2004; Kim *et al.*, 2015).

Moreover, Th9 cells could also indirectly inhibit tumor growth by secreting other cytokines. For example, Th9 cells could activate the immune cells such as NK, hypertrophy immune cells, or promote the differentiation of Th17 cells by secreting IL-21 to lead to apoptosis of tumor cells (Yang *et al.*, 2008; Humblin *et al.*, 2017). In line with these literatures, You *et al.* (2017) found that the increase of Th9 cells in the blood of breast cancer patients could promote the cytotoxicity of CD8^+^T cells through the expression of IL-21, and then participate in antitumor immunity. However, blocking IL-21 secretion could specifically inhibit the differentiation and function of Th9 cells, but does not directly affect the growth of tumor cells (Végran *et al.*, 2015), suggesting that the antitumor effect of Th9 cells is mainly achieved by their secreted cytokines.

Moreover, some other cytokines may also be involved in the antitumor effect of Th9 cells. For instance, TNF-α could enhance the antitumor effect of Th9 cells (Jiang *et al.*, 2019). Kim *et al.* (2019) found granulocyte/macrophage colony-stimulating factor (GM-CSF) could inhibit the differentiation of iTreg cells and GM-CSF-activated monocyte-derived DCs converted tumor-specific naive Th cells into Th9 cells, furthermore, restrained tumor growth by inducing antitumor CTLs in an IL9-dependent manner.

On the other hand, IL-9 secreted by Th9 cells could also promote the growth of some types of tumor cells, reflecting the complexity of its role in tumorigenesis. For instance, Tan *et al.* (2017) has reported that Th9 cells could promote tumor growth in hepatocellular carcinoma (HCC). Hoelzinger *et al.* (2014) further found that IL-9-deficient tumor-bearing mice survived longer than wild-type tumor-bearing mice. Furthermore, eliminated CD4^+^T cells and/or CD8^+^T cells from wild-type tumor-bearing mice could not affect the tumor growth of mice, indicating that Th9 cells could directly promote the growth of tumor cells by secreting IL-9. Moreover, IL-9 can also inhibit the apoptosis of tumor cells and promote their proliferation, as well as decrease the sensitivity of tumor cells to chemotherapeutic agents (Lv *et al.*, 2016).

Finally, Th9 cells could indirectly participate in tumorigenesis by affecting other immune cell functions. For example, Lv *et al.* (2016) found that IL-9 could enhance the immunosuppressive effect of Tregs and mast cells, thereby participating in the development of B lymphocytoma. In conclusion, these studies demonstrated the complexity of the roles of Th9 cells in tumorigenesis, but the exact mechanism remains to be further explored.

### Th9 cells and allergic inflammatory diseases

It is documented that in the condition of deficiency of Th9 cell transcription factors, such as PU.1, IRF4, and BATF, mice developed lower inflammation (Chang *et al.*, 2010; Staudt *et al.*, 2010; Jabeen *et al.*, 2013). Further studies showed that IL-9 secreted by Th9 cells could promote the expression of various chemokines in bronchial epithelial cells, which mediated the enrichment of immune cells, such as eosinophils and mast cells and aggravated respiratory inflammation (Sehra *et al.*, 2015). Moreover, IL-9 could also enhance IL-4-mediated B cell differentiation and IgE expression, and affected the proliferation of activated T cells and mast cells, thus promoting the occurrence of allergic inflammation (Dugas *et al.*, 1993; Koch *et al.*, 2017).

In addition, Th9 cells was also involved in the effect of some factors on allergic inflammation. Such as Schwartz *et al.* (2019) found that the vitamin A metabolite, retinoic acid, could inhibit Th9 differentiation, which was associated with human allergic inflammation. Therefore, immunotherapy against Th9 cells and its functional factor IL-9 may be one of the new treatment strategies for allergic inflammatory diseases (Dugas *et al.*, 1993; Hoppenot *et al.*, 2015; Reuter *et al.*, 2016; Gu *et al.*, Cao and 2017; Koch *et al.*, 2017; Jiang *et al.*, 2018).

Recently, Hamza *et al.* (2017) further found that the gene expression levels of IL-9 and PU.1 in patients with clinically specific dermatitis significantly increased, and positively correlated with the level of IgE. Moreover, the development of specific dermatitis could be analyzed by monitoring the changes of IL-9 and PU.1 gene levels (Ma *et al.*, 2014; Schlapbach *et al.*, 2014; Ciccia *et al.*, 2016), suggesting that Th9 cells not only participate in the development of allergic diseases, but may also be a new target for clinical monitoring of related clinical diseases.

### Th9 cells and systemic lupus erythematosus

Systemic lupus erythematosus (SLE) is a chronic autoimmune disease, which has unknown causes, multiple system organ involvement, multiple clinical and serum manifestations, mainly affecting women of childbearing age (Grover *et al.*, 2014). Ouyang *et al.* (2013) and Ciccia *et al.* (2016) found that the mRNA and protein level of serum IL-9 in patients with SLE were significantly higher than those in healthy controls. Moreover, the percentage of Th9 cells also significantly increased. Further analysis showed that the percentage of Th9 cells was significantly correlated with the disease activity index of SLE, and the level of serum IL-9 decreased obviously after treatment with glucocorticoid or methylprednisolone, suggesting that Th9 cells were not only involved in the occurrence of SLE, but could also be used as an important index of clinical efficacy. Interestingly, Elyaman *et al.* (2009) found that the suppressive activity of Treg cells from IL-9R^−/−^ mice exhibited a defect in comparison to their counterparts from WT mice, suggesting that IL-9/IL-9R pathway might have a protective role in SLE (Leng *et al.*, 2012). Therefore, IL-9 might be a potential therapeutic target for SLE (Yang *et al.*, 2015). However, the exact mechanism of the involvement of Th9 cells in the pathogenesis of SLE remains to be clarified.

## Conclusions and Prospects

In recent years, more and more important progress has been made in the study of Th9 cells, including the recent discovery that Treg cells could promote the production of Th9 cells by ligating glucocorticoid-induced TNFR-associated protein (GITR) (Kim *et al.*, 2015; Xiao, 2015), and the transcription factor IRF-1 was also involved in the differentiation of Th9 cells (Lu *et al.*, 2012). Additionally, Th9 cells might also play an important role in the pathogenesis of HIV (Gorenec *et al.*, 2016; Liwei *et al.*, 2017). Moreover, the changes in the proportion of Th9 cells in peripheral blood could also be used as biomarkers for the clinical treatment of cancer (Nonomura *et al.*, 2016; Lu *et al.*, 2018). Most recently, Vieira *et al.* (2019) found that butyrate treatment reduced lung inflammation by negatively regulating Th9 cells. Importantly, Micossé *et al.* (2019) further proposed that human Th9 cells is a subgroup of Th2 cells, which is called IL-9^+^ Th2 cells, distinguished from “conventional” Th2 cells based on their expression of the transcription factor peroxisome proliferator-activated receptor-γ (PPAR-γ). Meanwhile, Xue *et al.* (2019) found that the combination of IL-1β and IL-4 efficiently promoted the generation of IL-9-producing T cells (Th9^IL-4+IL-1β^), which were less exhausted when compared with classic Th9^IL-4+TGF-β^ cells and so on. These studies not only indicated the complexity of both differentiation of Th9 cells and plasticity of Th cells, but also demonstrated the important role of Th9 cells in the occurrence of related clinical diseases (as shown in Table 3).

**Table 3.** Th9 Cells and Related Diseases

*Diseases*	*Relationships*	*References*
Tumors	Inhibit tumor growth	Rumbo *et al.* (2004), Yang *et al.* (2008), Kim *et al.* (2015), Humblin *et al.* (2017), You *et al.* (2017), Jiang *et al.* (2019), Kim *et al.* (2019), Xue *et al.* (2019)
	Promote tumor growth	Hoelzinger *et al.* (2014), Lv *et al.* (2016), Tan *et al.* (2017)
	Biomarkers for the clinical treatment of cancer	Yang *et al.* (2015), Nonomura *et al.* (2016), Lu *et al.* (2018)
Allergic inflammatory diseases	IL-9 enhances inflammatory response	Dugas *et al.* (1993), Chang *et al.* (2010), Staudt *et al.* (2010), Jabeen *et al.* (2013), Ma *et al.* (2014), Schlapbach *et al.* (2014), Hoppenot *et al.* (2015), Ciccia *et al.* (2016), Reuter *et al.* (2016), Gu *et al.* (2017), Hamza *et al.* (2017), Koch *et al.* (2017), Jiang *et al.* (2018)
Systemic lupus erythematosus	Elevated serum level of IL-9 in patients	Elyaman *et al.* (2009), Leng *et al.* (2012), Ouyang *et al.* (2013), Ciccia *et al.* (2016)
HIV	Elevated expression level of PU.1 and number of Th9 cells	Gorenec *et al.* (2016), Liwei *et al.* (2017)

However, there are still many scientific issues that need to be further clarified in the future, such as: What is the exact networks, which are critical for the clarification of the value of CD4^+^T cell plasticity in the development of related clinical diseases, among Th9 cells and other different CD4^+^T cell subsets? Is there a relationship between the role of Th9 cells in different tumors and the microenvironment of the tumor? What is the mechanism of Th9 cells in the pathogenesis of autoimmune disease? and so on. In all, further elucidation of these scientific problems will provide a new idea for the study of the biological function of Th9 cells, the interpretation of complex immune cell network in immune response, the diagnosis, and treatment of clinical immune-related diseases.
